# Identification of Fish Species and Toxins Implicated in a Snapper Food Poisoning Event in Sabah, Malaysia, 2017

**DOI:** 10.3390/toxins13090657

**Published:** 2021-09-15

**Authors:** Ha Viet Dao, Aya Uesugi, Hajime Uchida, Ryuichi Watanabe, Ryoji Matsushima, Zhen Fei Lim, Steffiana J. Jipanin, Ky Xuan Pham, Minh-Thu Phan, Chui Pin Leaw, Po Teen Lim, Toshiyuki Suzuki

**Affiliations:** 1 Institute of Oceanography, Vietnam Academy of Science and Technology, 01 Cau Da, Nha Trang 650000, Vietnam; kyjapan2004@yahoo.com (K.X.P.); phanminhthu@vnio.org.vn (M.-T.P.); 2 Faculty of Marine Science and Technology, Graduate University of Science and Technology, Vietnam Academy of Science and Technology, 18 Hoang Quoc Viet, Ha Noi 100000, Vietnam; 3 Environment and Fisheries Applied Techniques Research Department, Fisheries Technology Institute, Japan Fisheries Research and Education Agency, 2-12-4 Fukuura, Kanazawa-ku, Yokohama 236-8648, Japan; aya.ue.ab@gmail.com (A.U.); huchida@affrc.go.jp (H.U.); rwatanabe@affrc.go.jp (R.W.); matsur@affrc.go.jp (R.M.); tsuzuki@affrc.go.jp (T.S.); 4 Bachok Marine Research Station, Institute of Ocean and Earth Sciences, University of Malaya, Bachok 16310, Kelantan, Malaysia; limzfrc@hotmail.com (Z.F.L.); cpleaw@um.edu.my (C.P.L.); ptlim@um.edu.my (P.T.L.); 5 Likas Fisheries Complex, Department of Fisheries Sabah, Kota Kinabalu 88400, Sabah, Malaysia; steffiana.jipanin@sabah.gov.my

**Keywords:** ciguatera fish poisoning (CFP), ciguatoxin-1B (CTX-1B), LC/MS, red snapper, Malaysia

## Abstract

In the coastal countries of Southeast Asia, fish is a staple diet and certain fish species are food delicacies to local populations or commercially important to individual communities. Although there have been several suspected cases of ciguatera fish poisoning (CFP) in Southeast Asian countries, few have been confirmed by ciguatoxins identification, resulting in limited information for the correct diagnosis of this food-borne disease. In the present study, ciguatoxin-1B (CTX-1B) in red snapper (*Lutjanus bohar*) implicated in a CFP case in Sabah, Malaysia, in December 2017 was determined by single-quadrupole selected ion monitoring (SIM) liquid chromatography/mass spectrometry (LC/MS). Continuous consumption of the toxic fish likely resulted in CFP, even when the toxin concentration in the fish consumed was low. The identification of the fish species was performed using the molecular characterization of the mitochondrial cytochrome c oxidase subunit I gene marker, with a phylogenetic analysis of the genus *Lutjanus*. This is the first report identifying the causative toxin in fish-implicated CFP in Malaysia.

## 1. Introduction

Ciguatoxins (CTXs) are well-known-marine toxins that can accumulate in various kinds of reef fish and marine invertebrates through the food chain and may cause human poisoning named ciguatera fish poisoning (CFP) by the consumption of contaminated fish [1,2,3]. In the coastal countries of Southeast Asia, there are extensive tropical and subtropical coral reefs, where ciguatoxic fishes are found [4]. Fish is a staple diet, and certain fish species are food delicacies to local populations or commercially important to individual communities [5,6,7]. 

Toxic dinoflagellates associated with CTXs are widely distributed in Malaysian waters [8,9,10]. Several CFP cases have been reported in Malaysia following the consumption of red snappers [11,12], with no confirmation of the causative toxins due to a lack of fish samples from poisoning cases. In late December 2017, there was a suspected CFP incident in Sabah, Malaysia, impacting a family of four members. They had a weekly routine of fish consumption as primary meals and had shown typical CFP-related symptoms. A detailed case report was presented by Lee et al. [13] explaining the cardiovascular, respiratory, neurological, and gastrointestinal symptoms experienced by the victims. 

In most cases, toxic fish samples are processed, cooked, and partly consumed; thus, morphological features of the causative fish are unidentifiable. A small proportion of the fish flesh, approximately 5 mm, is more than sufficient for accurate molecular identification. Primers targeting the mitochondrial cytochrome c oxidase subunit I (COI) gene marker is efficiently used for the identification of a variety of fish specimens, including fish eggs [14], exotic fish [15], and fish larvae [16]. The mitochondrial COI gene is a universally accepted fish DNA barcoding gene; for example, it has been used for the seafood fraud detection of grouper, *Epinephelus* Bloch [17]. In that particular study, the raw and cooked samples were successfully amplified using the primer set Fish F2 and R2 except for the deep-fry samples [17]. This has proven the efficiency of the primer set but with some exception of extreme cases where the DNA is denatured.

In the present study, the CFP-implicated fish specimens were collected from the patient’s home for analysis of the causative toxins and identification of the fish species. To our knowledge, there are no precautionary measures or monitoring of CTXs and their producers in Malaysia and other countries in the Southeast Asian region due to a lack of facilities. This study is thus important in the effort to raise awareness and to influence local authorities.

## 2. Results

### 2.1. Incident Case

A poisoning incident involving a family of four people (husband, wife, and two children) following the consumption of snapper occurred in late December 2017 at Kota Kinabalu, Sabah, Malaysia. The clinical case report has been described by Lee et al. [13]. Two fishes (approx. 2 kg body weight/each) were purchased from Lido Supermarket, Kota Kinabalu, Sabah, on two different days of a week. The fishes were cut into several small pieces (~50 g/piece) and stored in a freezer. On the first day of eating, symptoms such as muscle soreness, tiredness, and shortness of breath developed in all family members; however, the symptoms were mild, and unaware of the poisoning, they continued to consume the fish pieces as daily meals. After 6 days, reversed temperature sensation was recorded in two of the four patients (husband and wife). One victim, the wife, was hospitalized, and the symptoms persisted for six months, although there was no fatality.

### 2.2. Species Identification of Fish Samples

Sample selection was limited to the heads of the fishes as the other body parts including Flesh 1, 2, and 3 (Table 1) had been processed, cooked, and partially consumed, rendering individual fishes unidentifiable, with the exception of the heads. Table 1 showed the result of the identification of fish head samples collected in the CFP Malaysian incident using the molecular technique. Interestingly, the samples belonged to two snapper species, *Lutjanus bohar* Forsskål and *L. argentimaculatus* Forsskål, which are common commercial species in Malaysia as well as other Asian countries [18].

**Figure 1:**
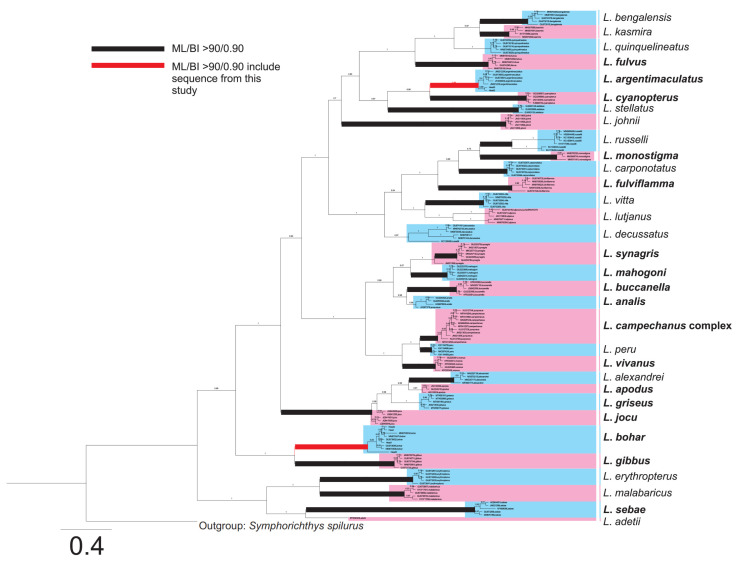
Phylogeny of the *Lutjanus* species based on COI gene sequences constructed using Maximum Likelihood (ML) and Bayesian Inference (BI). Species in boldface are fishes that have been implicated in CFP [18,19,20,21,22,23,24,25,26,27,28,29,30].

A total of 700 bp *COI* nucleotide sequences were successfully amplified and sequenced from all five fish head samples. The sequences obtained were compared with sequences from the NCBI Genbank nucleotide database using the BLASTn option. The samples “Head 1” and “Head 4” showed 100% identity to *Lutjanus bohar* with 100% query (accession: KF930067, MN870347, and MN870074). In addition, “Head 2” showed 97.92% identity to *L*. *bohar* with 100% query coverage. Both samples “Head 3” and “Head 5” showed identities of 100% and 99.68% to *Lutjanus argentimaculatus* (accession: MN243479, MN243478 and MG923374). The intra- and interspecific pairwise genetic distances of *L*. *bohar* and *L*. *argentimaculatus* were calculated using the sequences from the present study and of those from NCBI Genbank. The results showed that all the species were genetically distinct, with high interspecific variations, ranging from 15% to 16% (Supplementary Table S1) compared with the low intraspecific variations that ranged from 0% to 1.92% (Supplementary Tables S2 and S3). The *COI* phylogenetic reconstructions of Bayesian Inference (BI) and Maximum Likelihood (ML) revealed similar topologies, and BI was used as the core topology with both bootstrap values of ML and BI posterior probability presented (Figure 1). From the phylogeny, 25 of 33 species showed strong supports of ML bootstrap and BI posterior probability (>90/0.9); this included the two species of this study: *L*. *argentimaculatus*, with 94% ML bootstrap and 0.98 BI posterior probability, and *L*. *bohar*, with 95% ML bootstrap and 1.0 BI posterior probability. From all of the *Lutjanus* species analyzed, 18 species have been reported with ciguatera incidents following the records in FishBase [18] and in the literature [19,20,21,22,23,24,25,26,27,28,29,30].

### 2.3. Toxin in Fish Samples

**Figure 2:**
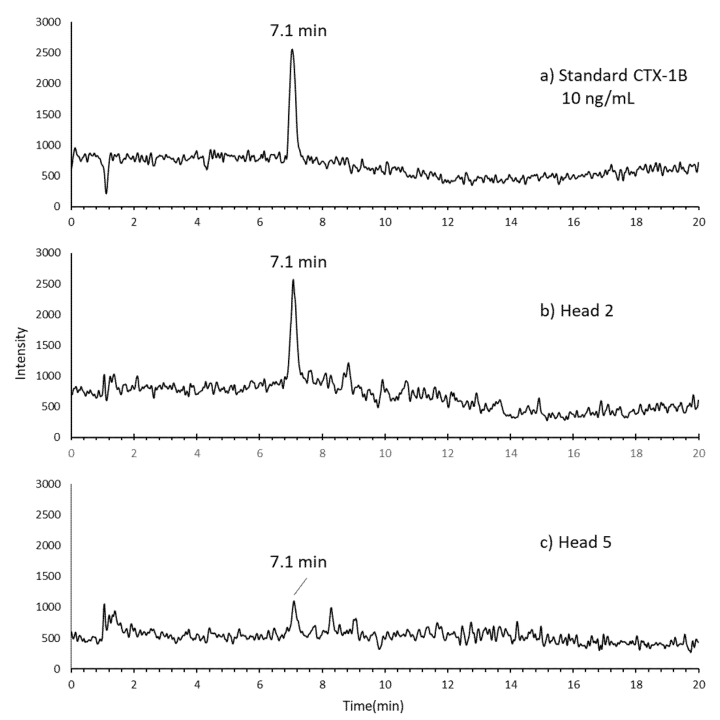
The SIM LC/MS chromatograms for [M+Na]^+^ of CTX-1B obtained from *L. bohar* implicated in CFP in Malaysia: (**a**) the reference standard of CTX-1B, (**b**) CTX-1B in *L. bohar* (sample “Head 2”), and (**c**) CTX-1B in *L.* *argentimaculatus* (sample “Head 5”).

With LC/MS analysis, among the nine samples collected from the Malaysian CFP incident, CTX-1B was detected in two samples: “Head 2” and “Head 5” (Table 1). The SIM LC/MS chromatograms of the standard CTX-1B, and the extracts of samples “Head 2” and “Head 5” are shown in Figure 2. The peak with the same retention time as the standard CTX-1B was detected in the extracts of both samples. Although all CTX analogues including 52-*epi*-54-deoxyCTX-1B and 54-deoxyCTX-1B found in the Pacific region [31,32,33] were monitored in the present study, they were not detected in the samples (Supplementary Figure S1). The CTX-1B concentration detected in sample “Head 2” of *L. bohar* was 0.38 ng/g tissue, which expressed 5.40 MU/100 g tissue of toxicity. Although CTX-1B was also detected in “Head 5”, the amount was below the LOQ (limit of quantification) (0.32 ng/g fish flesh) reported in our previous paper [31]. 

## 3. Discussion

CFP is generally reported after a clinical diagnosis based on the characteristic symptoms that occurred after eating reef fishes known to contain CTXs [34]. Neurological symptoms were observed in the patients from incidents that coincided with typical CFP symptoms reported from the Pacific Ocean [35,36]. Notably, identical reversed temperature sensation was reported in two victims, indicating that the Malaysian patients suffered more severe illness than those reported in previous CFP cases in Vietnam, where the levels of CTXs were higher in the implicated fish [31]. It could be due to continuous ingestion of the toxic fish pieces [37,38] although the amount of fish and CTXs consumed by the patients in the Vietnamese and Malaysian cases could not be determined.

CTX-1B, 52-*epi*-54-deoxyCTX-1B, and 54-deoxyCTX-1B were the most commonly observed toxins found in fish implicated in CFP cases in Pacific Ocean areas [31,32,33,39,40,41]. The presence of CTX-1B was confirmed using SIM LC/MS in one of the nine samples’ remnants in the Malaysian CFP case. Although there have been large numbers of reports and potential CFP cases in the Southeast Asian countries [4], these reports were mainly based on the clinical symptoms diagnosed in victims, and little is known about the fish species and the toxins involved in the CFP outbreaks [31]. In Malaysia, the first case of CFP was reported in April 2010 after consuming imported red snapper [11,12]. This was followed by incidents in May 2010, with 26 victims affected, and in September 2010, affecting 22 people from 5 families [13]. However, the causative toxin in the implicated snappers could not be identified due to insufficient remnants for toxin analysis.

Interestingly, among the nine samples analyzed, only two samples, “Head 2”, which belonged to *L. bohar* (which was previously designated as *L. sebae* in Lee et al. [13]), and “Head 5”, which belonged to *L. argentimaculatus*, contained CTX-1B, whereas no toxin was detected in the other seven (Table 1). The nine samples analyzed in our present study originated from two fish individuals. The concentration of CTX-1B in the *L. bohar* sample quantified in the present study (0.38 ng/g fish tissue; Table 2) was lower than that reported in Vietnam (0.9 and 3.7 ng/g fish tissue) [31]. It was also lower than that found in the Spanish mackerel *Scomberomorus commerson* from New South Wales (Australia) (1.0–1.3 μg/kg fish tissue) [42]. Continuous consumption of the toxic fish likely resulted in CFP, even when the toxin contents in the fish consumed was very low. The result indicated that *L. bohar* was the causative species in the CFP incident in Malaysia, as was the case in the Vietnam event [31], suggesting that the species could be a common source of CTXs in the region. Our results also indicated that CTX concentrations were largely different among fish specimens. *L. bohar* had higher levels of CTXs, while *L. argentimaculatus* had only trace levels; however, more data are needed to confirm. *L. bohar* has a strong association with CFP events and has been banned from consumption in some countries/regions [38].

Several genera of potentially toxic and harmful benthic dinoflagellates have been discovered in Malaysian waters, including *Gambierdiscus*, *Fukuyoa*, *Prorocentrum*, *Ostreopsis*, and *Amphidinium* [9,10,13,45,46]. Nonetheless, there is little knowledge on the distribution of CTXs in commercial fishes in Malaysia. Further investigation is therefore necessary for better risk assessment and management of CFP.

The molecular characterization of fish samples using COI gene sequences and the phylogeny, as revealed in this study, represents one of the most comprehensive phylogenetic coverages of *Lutjanus* species (33 species) compared with other phylogenetic studies on *Lutjanus* [47,48,49,50]; however, it could not cover every species of *Lutjanus* due to limited genetic information of the existing species (*n* = 180). The strongly supported phylogenetic positions of *L*. *bohar* and *L*. *argentimaculatus* showed the reliability of this molecular tool in the identification of CFP-related fish species. The high ML bootstrap and BI posterior probability of the majority of the species showed clear species distinction, with the exception of *L*. *camphenacus* and *L*. *purpureus*. These two species were often confused morphologically [51] and the molecular delineation using COI gene marker in this study appeared ambiguous. As the delineation could not be revealed in this study, the clade was grouped as a *L*. *camphecanus* complex. Even though *L*. *camphenacus* and *L*. *purpureus* showed synonymy, da Silva et al. [52] verified the delimitation of these two species using multilocus genetic data. In summary, the toxic fish was confirmed to be *L*. *bohar* using NCBI BLAST search, pairwise distance comparison, and the phylogeny. The species was previously identified as *L. sabae* by Lee et al. [13] based only on sequence similarity searching but did not consider further phylogenetic confirmation. In addition, an overview of the phylogenetic relationship of *Lutjanus* species and the implicated fish species that have been reported with CFP incidents is provided (Figure 1).

## 4. Materials and Methods

### 4.1. Sample Collection

Nine fish samples including five heads, three flesh, and one tail pieces that remained from the processed food in frozen condition were collected from the victim’s home on 15 December 2017 by the health officers of the Ministry of Health, Sabah Province, Malaysia. A small portion (<5 mg) of each sample was taken for DNA extraction, and the samples were lyophilized and sent to the Keylab on Food and Environmental Safety, Institute of Oceanography (Nha Trang City, Vietnam), Vietnam Academy of Science and Technology. The samples were kept at −20 °C prior to toxin analysis.

### 4.2. Reagents and Standards

High-performance liquid chromatography (HPLC)—(MeCN and MeOH) and analytical-grade solvents (acetone, MeOH, hexane, diethyl ether, and ethyl acetate) were purchased from Kanto Chemical Co., Inc. (Tokyo, Japan) and Fuji Film Wako Pure Chemical Co. (Osaka, Japan). Analytical-grade reagents (formic acid and ammonium formate) were purchased from Nacalai (Kyoto, Japan). Distilled water was passed through a Milli-Q water purification system (Millipore, Bedford, MA, USA) for the preparation of LC mobile phases. A reference standard of CTX-1B was kindly provided by Prof. Takeshi Yasumoto of Japan Food Research Laboratories. Reference standards of CTX-3C, CTX-4A, and CTX-4B were kindly provided by Dr. Mireille Chinain of Institut Louis Malardé.

### 4.3. Molecular Characterization of Fish Samples

#### 4.3.1. DNA Extraction and PCR

Genomic DNA of fish head samples was extracted using a TOYOBO MagExtractor Genome DNA purification kit (TOYOBO, Japan). Approximately 5 mg of fish tissue was transferred into a 1.5 mL microcentrifuge tube containing 90 μL of Proteinase K buffer, 5 μL of 10 mg/mL Proteinase K, and 5 μL of 10% SDS. The samples were incubated at 55 °C for 8 h. The samples were then centrifuged at 10,000 rpm for 5 min. A total of 100 µL of the supernatant was transferred into a new tube, and 750 μL of Lysis & Binding Solution was added. In the pretreated solution, 40 μL of a magnetic bead solution was added and mixed well for 10 min. Upon placement on the magnetic stand, the beads were captured, allowing for the process of washing with the wash solution and 70% ethanol. After the washing, 100 μL of sterilized water was added and mixed well for 10 min using a tube mixer. The DNA was used to amplify the mitochondrial cytochrome c oxidase subunit I (COI) gene. The primer set used was F2 (5′ CGT GTC AGT CAT GTG TCG CT 3′) and R2 (5′ CAA CAG CGT ATC GCT GGA AG 3′) [53]. The amplification condition was as follows: 5 min at 95 °C, followed by 32 cycles of 30 s at 95 °C, 30 s at 53 °C, 1 min at 72 °C, and a final extension of 10 min at 72 °C. Purified amplicons were directly sequenced for both strands using ABI 3730xl DNA analyzer (Applied Biosystems, Waltham, MA, USA).

#### 4.3.2. Taxon Sampling and Sequence Alignment

The sequences obtained were first checked using the NCBI Basic Local Alignment Search Tool (BLAST). The sequences were then translated into their corresponding peptide sequences using EMBOSS Transeq tool (EMBL-EBI) to examine the frameshift and presence of stop codons in the sequences. The protein sequences were further aligned using the PROMALS3D protein alignment program to check for sequence gaps. Only sequences confirmed as functional mitochondrial DNA (and not nuclear pseudogenes) were used. Our own sequences and related sequences of 157 taxa were then compiled into a FASTA file format. The nucleotide sequences were then aligned using the MUSCLE Multiple Sequence Alignment tool in EMBL-EBI. The multiple alignment file was manually trimmed using BioEdit sequence alignment editor v. 7.2.

#### 4.3.3. Pairwise Genetic Distance and Phylogenetic Analysis

The pairwise genetic distances between and among the species of *L. bohar* and *L. argentimaculatus* were calculated using Mega X [54]. The phylogenetic relationships of 33 species of *Lutjanus* were explored with Bayesian inference analyses (BI) and Maximum Likelihood (ML). The AIC model was generated using jModelTest 2.1.10 v.2 [55]. For Bayesian inference, the model values were set in Bayesian Evolutionary Analysis Utility (BEAUti) with MCMC of 10^7^ to generate an XML command file. The XML file was examined using Bayesian Evolutionary Analysis Sampling Trees (BEAST) [56] with a post-analysis performed using Tracer v.1.7.1 [57]. The burn-in value was set at 25%, and the phylogenetic tree was generated using MrBayes v.3.2 [58]. Maximum likelihood (ML) tree was reconstructed using RAxML (v. 8.4.10) using the transition model (TIM1) with gamma distribution and proportion of invariable sites, and 1000 bootstrap replications [59].

### 4.4. Extraction and Clean-Up of Fish Samples for LC/MS

The lyophilized fish tissues were hydrated overnight. The tissue was extracted with 150 mL of acetone, and the acetone extract was evaporated. The remaining residue was taken up in 40 mL of water and partitioned with 40 mL of diethyl ether. The diethyl ether layer, which contained the toxin, was collected and evaporated, and the residue was partitioned between MeOH/water (9:1, *v/v*) and hexane [60].

Sample clean-up was carried out according to Yogi et al. [32]. An extract equivalent to 5 g of flesh was dissolved in 2 mL of ethyl acetate/MeOH (9:1). The solution was passed through a Florisil cartridge (InertSep FL-PR, 500 mg), eluted with 2 mL of the same solvent, and then dried under nitrogen at 40 °C. The residue was dissolved in 3 mL of MeCN, applied to a PSA cartridge (InertSep PSA, 200 mg), and washed with 3 mL of MeCN, and the target toxins were eluted with 3 mL of MeOH. The eluate was dried and dissolved in 200 μL of MeOH for LC/MS analysis.

### 4.5. Quadrupole LC/MS

LC/MS analysis of toxins was carried with a model 1200 liquid chromatograph (Agilent, Palo Alto, CA, USA) coupled with a hybrid triple quadrupole/linear ion trap mass spectrometer Q Trap^TM^ 3200 (PE-SCIEX, Thornhill, ON, Canada). The LC conditions followed Suzuki et al. [33]. Separation was performed on an Agilent Poroshell 120 EC-C18 (100 × 2.1 mm i.d., 2.7 mm particle size) column maintained at 20 °C. Eluent A was 5 mM ammonium formate and 0.1% formic acid in water, and eluent B was methanol. A linear gradient elution from 80% to 95% B was performed over 10 min and then held for 10 min. The flow rate was 0.25 mL/min, and the injection volume was 5 µL. Instead of multiple reaction monitoring (MRM) selecting [M+Na]^+^ in both the target parent and the fragment ions in Q1 and Q3 [31,32,33], selected ion monitoring (SIM) selecting [M+Na]^+^ in Q1 with a dwell time of 63 ms for each analogue was applied [31]. Ion spray voltage (IS) and temperature in IS (TEM) were set at 5500 V and 500 °C. Ion source gas 1 (GS1) and GS2 were 80 and 30, respectively. The declustering potential (DP) and entrance potential (EP) were 400 and 12 V, respectively. The other MS parameters are the same as described previously [32].

## Figures and Tables

**Table 1:** Species identification of implicated fish in the poisoning event in Sabah, Malaysia, 2017.

Sample Name	Condition of Sample	Fish Species
Head 1	Uncooked, frozen	*Lutjanus bohar*
Head 2	Uncooked, frozen	*Lutjanus bohar*
Head 3	Uncooked, frozen	*Lutjanus* *argentimaculatus*
Head 4	Uncooked, frozen	*Lutjanus bohar*
Head 5	Uncooked, frozen	*Lutjanus* *argentimaculatus*
Flesh 1	Cooked, partially eaten	*Lutjanus*
Flesh 2	Cooked, partially eaten	*Lutjanus*
Flesh 3	Cooked, partially eaten	*Lutjanus*
Tail 1	Cooked	*Lutjanus*

**Table 2:** Ciguatoxin-1B level in fish samples implicated in a CFP event in 2017 in Sabah, Malaysia.

Sample Name	Fish Species	CTX-1B (ng/g)	Total Toxicity (MU/100g Fish Tissue) Estimated by LC/MS *^1^
Head 1	*Lutjanus bohar*	ND *^2^	-
Head 2	*Lutjanus bohar*	0.38	5.40
Head 3	*Lutjanus argentimaculatus*	ND	-
Head 4	*Lutjanus bohar*	ND	-
Head 5	*Lutjanus argentimaculatus*	Trace *^3^	-
Flesh 1	*Lutjanus*	ND	-
Flesh 2	*Lutjanus*	ND	-
Flesh 3	*Lutjanus*	ND	-
Tail 1	*Lutjanus*	ND	-

*^1^ Total toxicity (MU/g) = CTX-1B content (ng/g)/7 (ng/MU); the conversion factor (ng/MU) of CTX-1B is 7 [43,44]. *^2^ Not detected by LC/MS. *^3^ Below the LOQ (Limit of Quantification).

## Data Availability

Not applicable.

## Supplementary Materials

The following are available online at https://www.mdpi.com/article/10.3390/toxins13090657/s1, Figure S1: The SIM LC/MS chromatograms for [M+Na]+ of standard CTX-3C (above) and CTX-4A/4B (below); Table S1: Interspecific pairwise genetic distances of *Lutjanus bohar* and *L. argentimaculatus*, Table S2: Intraspecific pairwise genetic distances of *Lutjanus argentimaculatus*; Table S3: Intraspecific pairwise genetic distances of *Lutjanus bohar*.
