# Supplementary material for: Identification of Fish Species and Toxins Implicated in a Snapper Food Poisoning Event in Sabah, Malaysia, 2017
Source: Toxins (Basel). 2021 Sep 15;13(9):657. doi: 10.3390/toxins13090657 (PMC8470750; doi:10.3390/toxins13090657)
Supplement: Supplementary file 1 [file toxins-13-00657-s001.zip › toxins-1356070-supplementary.pdf]

molecules

MDPI

Supplementary Materials: Identification of Fish Species and Toxins Implicated in a Snapper Food Poisoning Event in Sabah, Malaysia, 2017

Ha Viet Dao, Aya Uesugi, Hajime Uchida, Ryuichi Watanabe, Ryoji Matsushima, Zhen Fei Lim, Steffiana J. Jipanin, Ky Xuan Pham, Minh-Thu Phan, Chui Pin Leaw, Po Teen Lim and Toshiyuki Suzuki

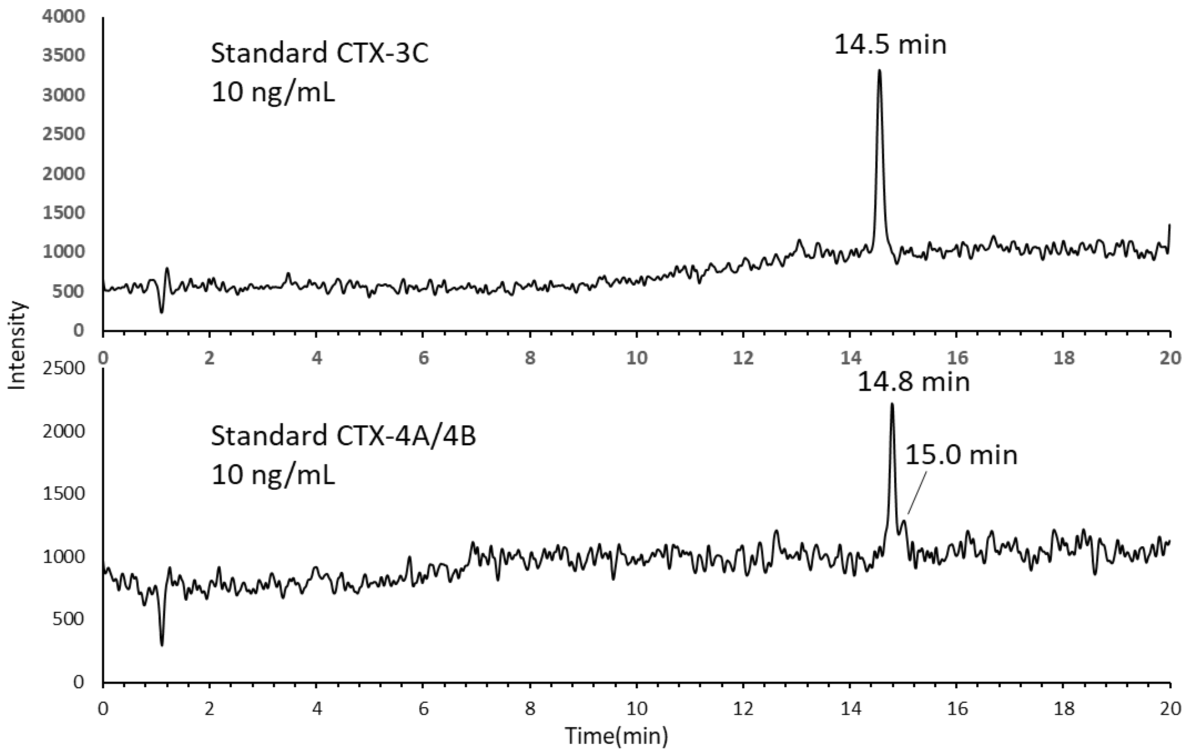

Figure S1. The SIM LC/MS chromatograms for $[M+Na]^+$ of standard CTX-3C (above) and CTX-4A/4B (below).

Table S1. Intraspecific pairwise genetic distances of *Lutjanus argentimaculatus*.

	GU673900Largenti-maculatus	Head3	GU673901Largenti-maculatus	Head5	JF493820Largenti-maculatus	JN021224Largenti-maculatus
GU673900Largentimaculatus						
Head3	0.003273322					
GU673901Largentimaculatus	0.001636661	0.00491				
Head5	0.003273322	0	0.00491			
JF493820Largentimaculatus	0.001636661	0.00491	0.003273	0.00491		
JN021224Largentimaculatus	0.001636661	0.00491	0.003273	0.00491	0.003273322	
JN021223Largentimaculatus	0.001636661	0.00491	0.003273	0.00491	0.003273322	0.003273

Table S2. Intraspecific pairwise genetic distances of *Lutjanus bohar*.

	**MN870347Lbohar**	**Head4**	**Head1**	**GU673902Lbohar**	**Head2**	**GU673839Lbohar**	**MN870358Lbohar**
MN870347Lbohar							
Head4	0.0000000000						
Head1	0.0000000000	0.0000000000					
GU673902Lbohar	0.0000000000	0.0000000000	0.0000000000				
Head2	0.0196721311	0.0196721311	0.0196721311	0.0196721311			
GU673839Lbohar	0.0016366612	0.0016366612	0.0016366612	0.0016366612	0.0213114754		
MN870358Lbohar	0.0016366612	0.0016366612	0.0016366612	0.0016366612	0.0213114754	0.0000000000	
MN870523Lbohar	0.0065466448	0.0065466448	0.0065466448	0.0065466448	0.0262295082	0.0081833061	0.0081833061
